# Stress Analysis in Conversion Total Hip Arthroplasty: A Finite Element Analysis on Stem Length and Distal Screw Hole

**DOI:** 10.3390/jcm14010106

**Published:** 2024-12-28

**Authors:** Koshiro Shimasaki, Tomofumi Nishino, Tomohiro Yoshizawa, Ryunosuke Watanabe, Fumi Hirose, Shota Yasunaga, Hajime Mishima

**Affiliations:** Department of Orthopaedic Surgery, Institute of Medicine, University of Tsukuba, 1-1-1, Tennodai, Tsukuba 305-8575, Ibaraki, Japan; koshiro19881020@tsukuba-seikei.jp (K.S.); tyoshizawa@tsukuba-seikei.jp (T.Y.); ryuwatanabe@tsukuba-seikei.jp (R.W.); f.ochiai.0023@tsukuba-seikei.jp (F.H.); syasunaga@tsukuba-seikei.jp (S.Y.); hmishima@tsukuba-seikei.jp (H.M.)

**Keywords:** osteoporosis, femoral trochanteric fracture, conversion total hip arthroplasty, periprosthetic fracture, stress distribution

## Abstract

**Background/Objectives:** Proximal femoral fractures are particularly common in older adults, and cases requiring conversion to total hip arthroplasty may arise because of treatment failure or osteoarthritis. Fractures around the distal screw removal holes can be problematic. This study aimed to analyze the relationship between stem length and femoral stress distribution to determine the optimal stem length. **Methods:** A finite element analysis simulation was conducted using pre-existing femoral computed tomography data, an intramedullary nail, and three types of stems of varying lengths. Loads simulating normal walking and stair climbing were applied, and the average and maximum equivalent stresses were measured on both the medial and lateral sides of the distal screw removal hole for each stem length. Statistical analysis was then performed to evaluate the stress distributions. **Results:** The average stress around the distal screw removal hole tended to decrease as stem length increased. The maximum stress was significantly lower with the 160-mm stem, which provides a 40-mm bridging length, compared to the 120-mm and 130-mm stems, where the stem tip aligned with or only slightly extended past the distal screw removal hole (bridging lengths of 0 mm and 10 mm, respectively). **Conclusions:** In conversion hip total arthroplasty following proximal femoral fractures, using a sufficiently long stem can help avoid stress concentration around the distal screw removal hole, thereby potentially reducing the risk of periprosthetic fractures.

## 1. Introduction

Proximal femoral fractures, which are particularly common in older adults, are frequently managed surgically using intramedullary nails, especially in those with unstable fracture patterns, as indicated by the American and British guidelines [1,2]. However, complications such as cut-out, nonunion, and pseudarthrosis, or conditions like femoral head necrosis and osteoarthritis, may necessitate salvage surgery, specifically conversion to total hip arthroplasty (cTHA) [3,4,5,6]. Compared to primary THA, cTHA is more complex, complicated by proximal femur nonunion, bone defects, poor bone quality, reduced offset, bone sclerosis around prior hardware removal sites, and challenges in fracture fixation and stem selection [7,8,9,10,11].

Consequently, cTHA involves longer surgery times, greater blood loss [7,12,13,14,15], and a higher risk of complications, including dislocation (11.4%), periprosthetic fractures (6.2%), and infection (3.8%) [8,15,16]. The 1-year mortality rate post-cTHA has also been reported at 13.6% [13,14,17,18,19,20]. With the growing older adult population and increased incidence of femoral fractures, the demand for cTHA and attention to its associated risks are expected to increase.

Periprosthetic fractures during cTHA are classified as intraoperative or postoperative. Intraoperatively, fractures often occur around the proximal femur, such as in the greater trochanter or calcar region [7,8,9,10,11], owing to surgical stress from reaming or stem insertion, particularly in osteoporotic or previously deformed bones, leading to iatrogenic fractures [8,21]. Postoperatively, fractures are observed not only in the proximal femur but also around the distal screw removal holes [14,22,23,24], often resulting from long-term osteolysis or stem loosening [25], or in the short term from stress concentration at the distal stem [26,27], often occurring without significant trauma [28,29].

Cortical holes left by previous screws should ideally be plugged during surgery whenever possible. This is because these holes can lead to inadequate cement pressurization when using a cemented stem and serve as potential sites for stress concentration, increasing the risk of femoral fracture when using a cementless stem [8].

In terms of stem length, short stems have the advantage of preserving bone stock and achieving a more physiological distribution of femoral stress. However, in patients with poor bone quality and limited bone stock, there is concern about the risk of periprosthetic fractures around the stem due to increased stress on the bone. On the other hand, long stems are expected to reduce the risk of localized stress concentration through higher stress distribution effects and greater stiffness than short stems. Nevertheless, stress shielding in the proximal region can be a significant issue, and if the load is not transmitted through the proximal femur, proximal bone loss is inevitable [30,31,32].

While some reports argue that extensive bridging over screw removal holes is unnecessary [33], many surgeons favor the use of long revision stems [8,11,34,35,36,37,38], particularly with cortical defects, and recommend a bridging length of approximately two femoral diameters (approximately 40 mm) [39,40,41]. However, the optimal bridging length and the effects of stem length on the stress distribution around screw removal holes remain under-researched, with no consensus on best practices.

Currently, there are reports indicating that there is no difference in clinical outcomes for cTHA depending on the stem used or techniques employed for bone fixation. Therefore, from the perspective of femoral stress distribution, it is considered practical to use either a cementless long stem or a cemented stem in clinical practice. From the perspective of risks, such as insufficient cement injection pressure due to multiple implant removal holes and the risk of BCIS (Bone Cement Implantation syndrome), the use of cementless stems is appealing.

We, therefore, aimed to analyze the stress distribution around the distal screw removal hole during cTHA following proximal femoral fractures and determine the optimal stem length. Simulations were conducted using finite element analysis (FEA) with stems of varying lengths. Our hypothesis was that the stress concentration would peak when the stem tip was aligned with the distal screw removal hole. Furthermore, considering that a bridging length of at least twice the femoral diameter is recommended for fractures or large cortical defects [39,40,41], we hypothesized that a length of 1.5 × the femoral diameter may significantly reduce stress around the screw removal hole.

## 2. Materials and Methods

Informed consent was obtained from the patient for the use and publication of the data. This analytical observational study was approved by the Ethics Review Committee of our institution (approval code: H27-041). This analytical observational study utilized existing computed tomography (CT) and femoral implant Standard Triangulated Language (STL) data to conduct FEA simulations. We then aimed to analyze the stress distribution in the femur.

Before starting this study, we calculated the required sample size using the statistical power analysis software, G*Power (version 3.1.9.7; University of Düsseldorf, Düsseldorf, Germany). Assuming an effect size of 0.25, a significance level of 0.05, a power of 0.8, and three groups for the parametric test, the minimum required sample size was determined to be 28. Based on this, we decided to analyze 30 cases (30 limbs) of femoral CT data. The population from which the sample data were drawn consisted of 100 female patients aged 75 or younger who underwent their first total hip arthroplasty at our institution between October 2021 and September 2024, all classified as Dorr Type B [42]. Patients with a history of femoral osteotomy or severe deformities, such as Perthes-like deformities, were excluded. Thirty patients (30 limbs) were randomly selected for analysis to minimize selection bias and improve statistical power, thereby enhancing the reliability and validity of the study results.

Femoral CT data were obtained from the unaffected side. Because CT imaging conditions were standardized during the study period, data quality was sufficiently ensured, and selection bias related to the period was negligible. One of the evaluation methods for femoral morphology, the Dorr classification, involves a subjective assessment. In this study, three experienced orthopedic surgeons independently evaluated the preoperative plain radiographs and classified the femoral morphology as Type A, B, or C. The intraclass correlation coefficient (ICC) was used to assess inter-rater reliability, yielding an ICC of 0.80 (95% CI: 0.72–0.88), demonstrating high consistency among the raters. Therefore, we confirmed that the Dorr classification used in this study has sufficient reliability.

CT data used in this study were obtained using a 256-slice multidetector CT scanner (Brilliance iCT; Philips Healthcare, Cleveland, OH, USA). The femur was scanned from the pelvis to both knee joints, along with a bone density phantom (QRM-BDC/3 Phantom, QRM Quality Assurance in Radiology and Medicine GmbH, Möhrendorf, Germany) under conditions of 120 kV/166 mAs and 1 mm slice thickness.

The FEA analysis software used was MECHANICAL FINDER (MF, version 13.0, Extended Edition, Research Center of Computational Mechanics, Tokyo, Japan), which constructs a heterogeneous material model based on bone density derived from CT values and allows for detailed stress and strength analysis. First, the CT data were imported into the MF, and a three-dimensional (3D) femoral model of the unaffected side was constructed. Next, a femoral model was created after screw removal (removal model). In this study, a 3D–3D registration was performed on the healthy femur model and the STL data of the intramedullary nail in MF, and the material properties of the nail were set to “unused” to replace the nail with a void, thus recreating the femur after screw removal.

The intramedullary nail used was the Trochanteric Fixation Nail Advanced (TFNA) Proximal Femoral Nailing System (Depuy Synthes, Raynham, MA, USA), represented by φ10 mm × 200 mm/130° STL data. The distal screw was 5 mm long and designed to be 135 mm from the proximal end of the nail. The screw was inserted into the 3D femoral model such that the tip-apex distance was <20 mm, with the distal screw fixed statically at a diameter of 5 mm. The stem used was a Universia stem (Teijin Nakashima Medical, Okayama, Japan) #11, high offset, in the STL format. The Universia stem is designed to fit the femoral morphology of the Japanese population and is a cementless stem coated with hydroxyapatite on all surfaces [43].

**Figure 1:**
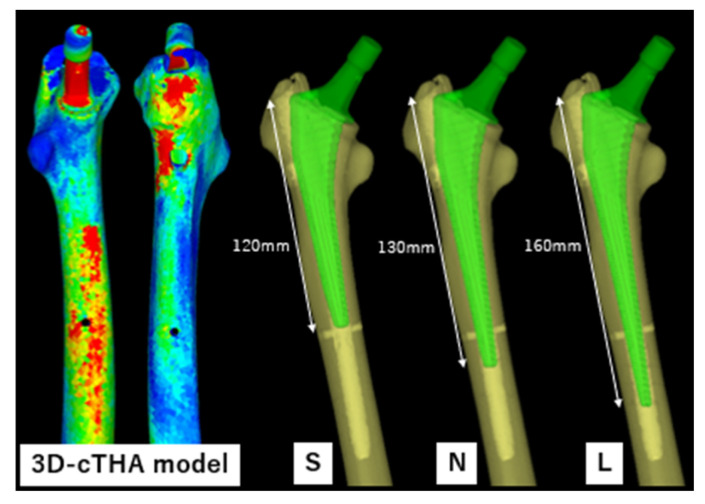
3D-cTHA model, constructed by inserting a stem into a 3D femoral model after intramedullary nail removal. Three types of stems were used: a short stem (S, 120 mm) with its tip at the same level as the distal screw extraction hole; a normal stem (N, 130 mm) bridging 10 mm beyond the hole; and a long stem (L, 160 mm) bridging 40 mm.

The STL data from the Universia stem were inserted into the removal model to create a cTHA model (Figure 1). The stems were divided based on their length into three groups: (1) short (120 mm stem, hereafter referred to as the S group); (2) normal (130 mm stem, N group); and (3) long (160 mm stem, L group) (Figure 1). In clinical practice, the 130 mm normal stem is the only one used, whereas the short and long stems were newly constructed virtual stems for this simulation. The long stem was selected based on previous studies that recommended a bridging length of approximately 40 mm for fractures or large cortical bone defects [39,40,41], and a stem length of 160 mm was used for verification.

**Figure 2:**
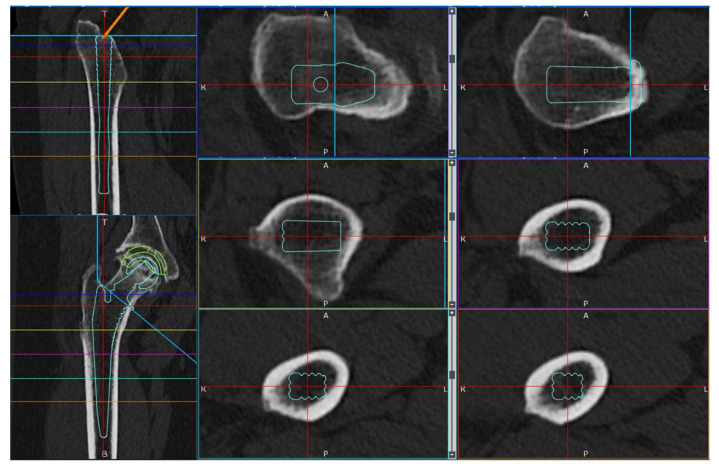
Preparation for cTHA. Preoperative planning was performed using the 3D preoperative planning software, ZedHip (version 17.0.0; Lexi Co., Ltd., Tokyo, Japan).

Simulations were conducted under a load to analyze the femoral stress distribution for each stem length. The femoral neck osteotomy and stem placement positions were initially planned using the 3D preoperative planning software, ZedHip (version 17.0.0; Lexi Co., Ltd., Tokyo, Japan) (Figure 2) and were faithfully reproduced in the MF.

### 2.1. Material Parameters

**Figure 3:**
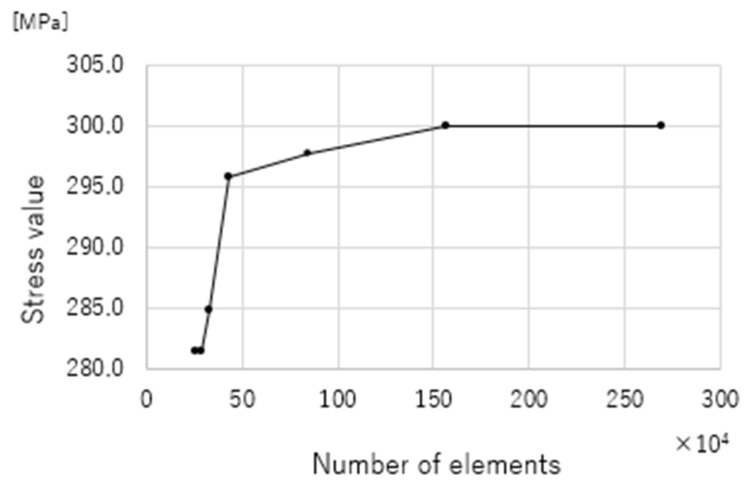
Mesh convergence test. The optimal mesh size was determined by confirming the conditions under which the stress variation remained below 1%. This choice balances the analysis precision and computational efficiency, allowing high accuracy while minimizing the calculation time.

Solid elements with 4-node tetrahedral elements were used in this study. A shell element with a thickness of 0.001 mm, which did not influence the strength, was applied to the bone surface. A mesh convergence test was conducted to determine the mesh size (Figure 3).

To determine an appropriate mesh size for the finite element analysis, a mesh convergence test was conducted. Several mesh sizes were evaluated, and the resulting stress values were compared to identify the point at which further refinement caused a change of less than 1%. Based on these results, an optimal mesh size was selected to balance computational efficiency and solution accuracy. This approach ensured that the analysis provided reliable results while minimizing unnecessary computational costs.

An inhomogeneous material model was used for the bones. Young’s modulus was calculated from the bone mineral density (BMD, ρ [g/cm^3^]) based on the CT values (Hounsfield Units: HU) using a linear relationship [44,45]. Subsequently, the values were estimated using Keyak’s predictive transformation formula and incorporated into the model [46]. Poisson’s ratio was set as 0.40 [44,46]. The stem was modeled using a homogeneous material model with the material properties of a titanium alloy (Ti-6Al-4V), Young’s modulus of 109 GPa, and Poisson’s ratio of 0.28 [44,46] (Table 1).

### 2.2. Loading and Boundary Conditions

**Figure 4:**
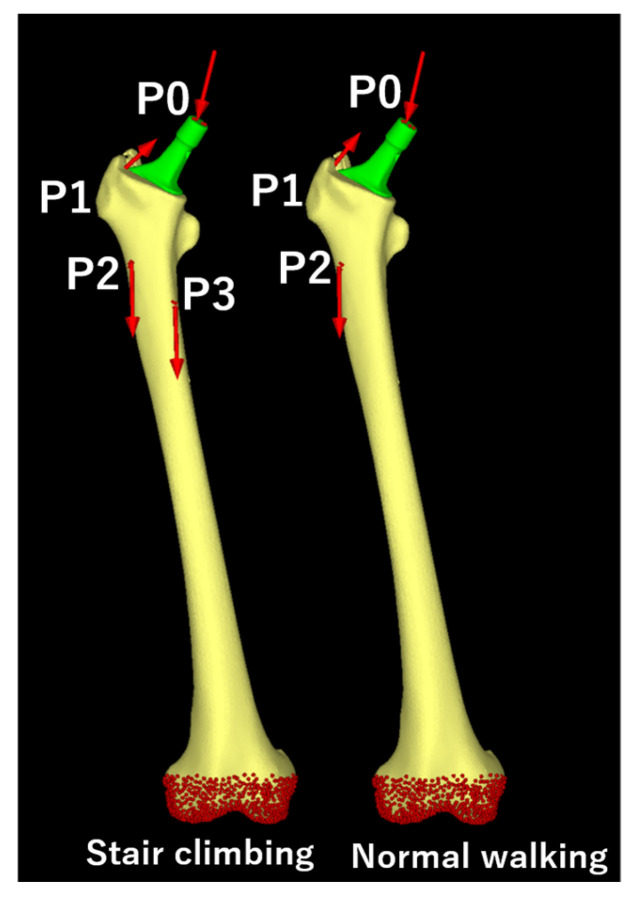
Loading conditions. Loading points and fixation sites for simulated normal climbing and stair climbing. P0, hip contact point; P1, a combined force of the abductor muscles and iliotibial band; P2, action point of the vastus lateralis; P3, action point of the vastus medialis. The distal femur was fully constrained under both conditions.

The loading conditions were based on the maximum load during daily activities. For this study, maximum loads during “normal walking” and “stair climbing” were adopted, based on previous reports [47,48]. The loads are shown in Table 2 and Figure 4, and the magnitude of each vector was determined based on the weight of the patient. The boundary conditions were set for full restraint at the distal femur (Figure 4), and the contact condition between the femur and stem was defined with a friction coefficient of 0.49 [49].

### 2.3. Static Structural Analysis

The load was applied linearly, and an elastic analysis was performed. The calculations were performed using linear static analysis.

### 2.4. Data Collection and Candidate Predictors

Patient background data included age (years), height (m), weight (kg), BMI (kg/m^2^), femoral neck BMD (g/cm^2^), and Canal Flare Index (CFI) [50]. Preoperative measurements of age, height, weight, BMI, and BMD were assessed using a Hologic Discovery A scanner (Hologic Inc., Bedford, MA, USA). Additionally, the parameters of the constructed cTHA model, such as the stem anteversion angle (°), bridging length (mm), and distal screw length (mm), were measured. To ensure intra-observer reliability, each parameter was measured three times under the same conditions, and the average of these measurements was used for the final data. All data were expressed as mean ± standard deviation (SD).

### 2.5. Outcomes

**Figure 5:**
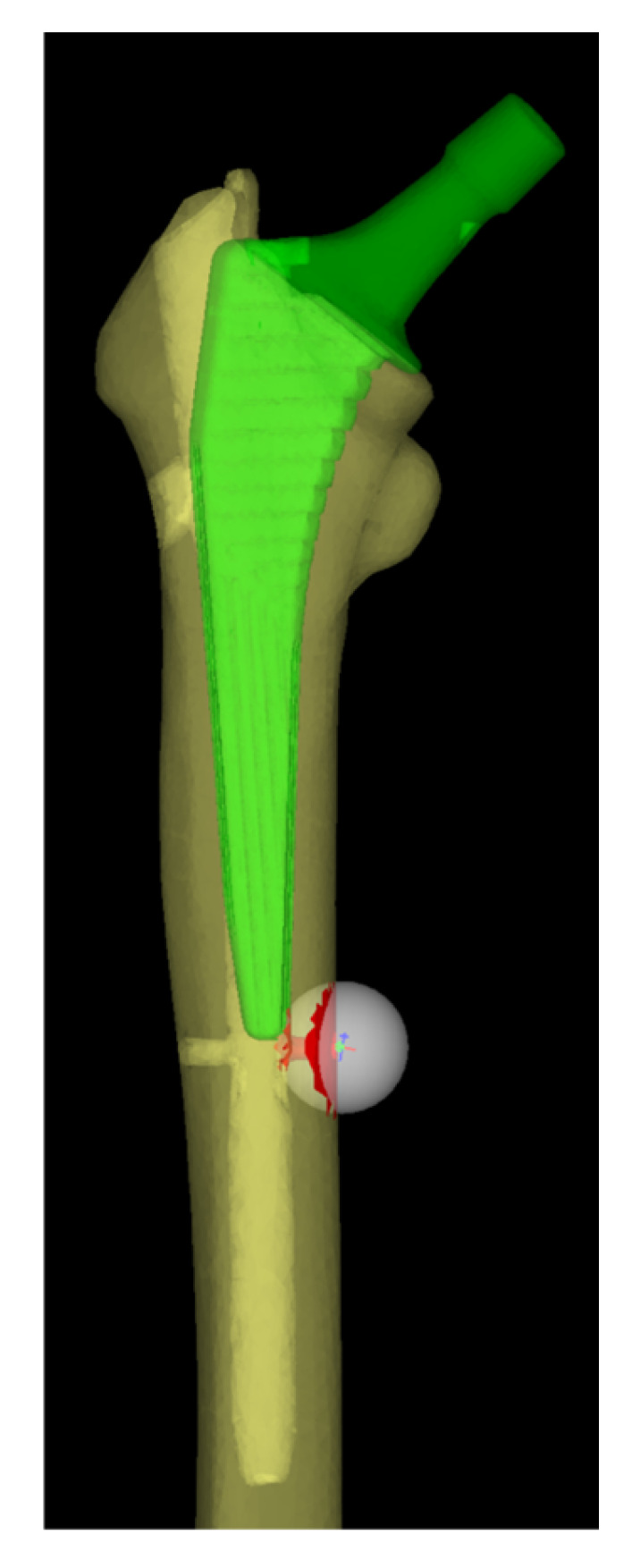
Regions of interest for stress value calculations. The target area of investigation was defined as the shell elements of the bone surface within a spherical region with a 10-mm radius centered on the distal screw removal hole.

Simulations were performed for the S, N, and L groups during “normal walking” and “stair climbing”, and a linear analysis was used to calculate the average and maximum stress values on the medial and lateral sides of the distal screw removal hole. The study area was defined as the bone surface shell elements within a 10-mm radius sphere centered around the distal screw removal hole (Figure 5). The equivalent stress [MPa] was used, and all data were expressed as mean (95% CI).

### 2.6. Statistical Analysis

Statistical analysis of the maximum equivalent stress around the distal screw removal hole was performed using SPSS Statistics version 29 (IBM Corp., Armonk, NY, USA). The normality of the data was assessed using the Shapiro–Wilk test. A significance level of α = 0.05 was set; if the *p*-value exceeded 0.05, the data were considered normally distributed, and an appropriate statistical test to compare the three groups was selected.

## 3. Results

### 3.1. Flow Chart of Patients

**Figure 6:**
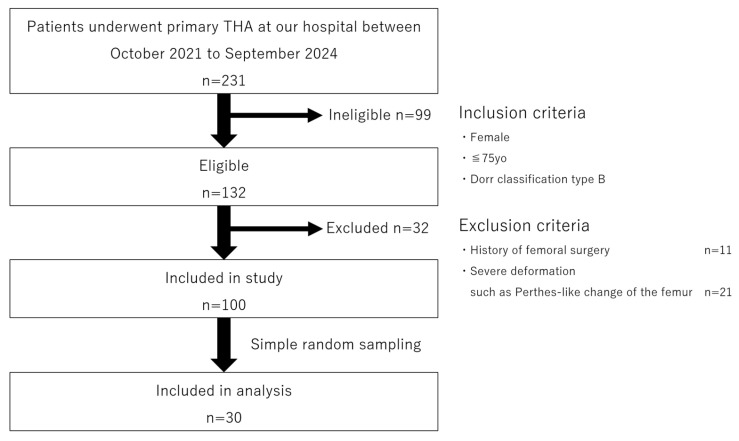
Flowchart of study data selection, including patient recruitment and exclusion criteria.

Patient flow in this study is shown in Figure 6.

First, out of a total of 231 patients who underwent THA at our institution between October 2021 and September 2024, we identified 132 cases involving women aged 75 or younger with Dorr classification type B. Among these, 11 cases with a history of femoral surgery and 21 cases with severe deformities such as Perthes-like deformities were excluded, leaving 100 eligible cases. From these 100 cases, 30 were selected using a simple random sampling method, and the contralateral femoral CT data from these patients were used for analysis.

### 3.2. Characteristics of Patients and 3D-cTHA Model

The characteristics of the patients and the 3D-cTHA model used in this study are summarized in Table 3.

### 3.3. Analysis

**Figure 7:**
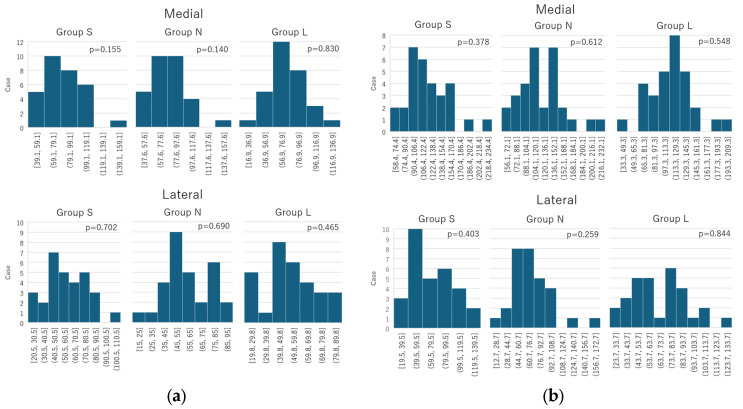
Histogram of the normality test performed using the Shapiro–Wilk test. (**a**) Normal walking; (**b**) Stair climbing. The collected data followed a normal distribution under both the normal and stair-climbing conditions.

The maximum equivalent stress values at the medial and lateral sides of the distal screw removal hole during “normal walking” and “stair climbing” for the three groups (S, N, and L) were confirmed to follow a normal distribution. The normality tests for each group showed: “normal walking” (medial side: S group, W = 0.949, *p* = 0.155; N group, W = 0.947, *p* = 0.140; L group, W = 0.980, *p* = 0.830; lateral side: S group, W = 0.976, *p* = 0.702; N group, W = 0.975, *p* = 0.690; L group, W = 0.967, *p* = 0.465); “stair climbing” (medial side: S group, W = 0.963, *p* = 0.378; N group, W = 0.973, *p* = 0.612; L group, W = 0.970; *p* = 0.548; lateral side: S group, W = 0.965, *p* = 0.403; N group, W = 0.957, *p* = 0.259; L group, W = 0.981, *p* = 0.844) (Figure 7).

Based on these results, repeated measures analysis of variance (ANOVA) was used to compare the three groups. The Bonferroni correction was applied for post-hoc multiple comparisons, and pairwise differences between all three groups were evaluated.

### 3.4. Mean Equivalent Stress Around the Distal Screw Removal Hole

**Figure 8:**
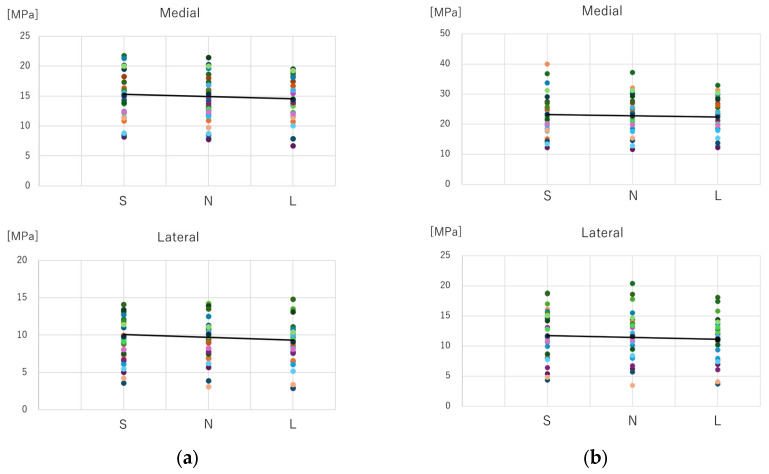
Comparison of average values of equivalent stress. (**a**) Normal walking; (**b**) Stair climbing. Under both normal walking and stair climbing conditions, the average stress around the distal screw removal hole tended to decrease as the stem length increased.

A scatter plot of the mean equivalent stress values at the distal screw removal hole for different stem lengths and fitted lines is shown in Figure 8. Both during “normal walking” and “stair climbing”, the mean equivalent stress decreased with increasing stem length.

### 3.5. Maximum Equivalent Stress Around the Distal Screw Removal Hole

#### 3.5.1. Normal Walking

a. Comparison between groups

**Figure 9:**
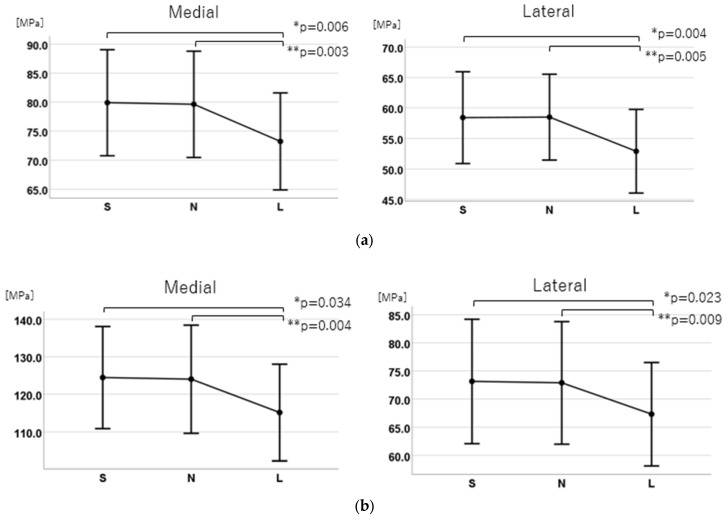
Comparison of the maximum equivalent stress values. (**a**) Normal walking; (**b**) Stair climbing. In both the normal walking and stair-climbing conditions, a significant difference was observed between Groups S and L (*), as well as between Groups N and L (**). However, no significant difference was found between Groups S and N.

For the medial side, the stress was 79.9 (95% CI: 70.8–89.0) MPa for the S group, 79.6 (95% CI: 70.5–88.8) MPa for the N group, and 73.2 (95% CI: 64.9–81.6) MPa for the L group. For the lateral side, it was 58.4 (95% CI: 50.9–65.9) MPa for the S group, 58.5 (95% CI: 51.5–65.5) MPa for the N group, and 52.9 (95% CI: 46.0–59.8) MPa for the L group. Both medial and lateral sides showed significant main effects in the analysis of variance (medial side: F (2, 58) = 9.940, *p* < 0.001, η^2^ = 0.26; lateral side: F (2, 58) = 8.311, *p* < 0.001, η^2^ = 0.22) (Figure 9).

b. Post-hoc multiple comparisons

Significant differences were found between the S and L groups and the N and L groups on both the medial and lateral sides (S vs. L: medial, *p* = 0.006; lateral, *p* = 0.004; N vs. L, medial *p* = 0.003, lateral *p* = 0.005). On the medial side, the stress in the S group was 6.67 MPa higher than that in the L group (95% CI: 1.69–11.64), and 6.40 MPa higher than that in the N group (95% CI: 1.96–10.83). On the lateral side, the S group showed 5.52 MPa higher stress than the L group (95% CI: 1.53–9.50) and 5.50 MPa higher than the N group (95% CI: 1.48–9.52). No significant differences were found between the S and N groups (medial and lateral: *p* > 0.99) (Figure 9).

#### 3.5.2. Stair Climbing

a. Comparison between groups

For the medial side, the stress was 124.5 (95% CI: 110.84–138.06) MPa for the S group, 124.0 (95% CI: 109.6–138.4) MPa for the N group, and 115.1 (95% CI: 102.3–128.0) MPa for the L group. For the lateral side, it was 73.1 (95% CI: 62.1–84.2) MPa for the S group, 72.9 (95% CI: 62.0–83.8) MPa for the N group, and 67.3 (95% CI: 58.2–76.5) MPa for the L group. Both medial and lateral sides showed significant main effects in the analysis of variance (medial side: F (2, 58) = 7.220, *p* = 0.004, η^2^ = 0.20; lateral side: F (2, 58) = 6.110, *p* = 0.004, η^2^ = 0.17) (Figure 9).

b. Post-hoc multiple comparisons

Significant differences were found between the S and L groups and the N and L groups on both the medial and lateral sides (S vs. L: medial, *p* = 0.034; lateral, *p* = 0.023; N vs. L, medial *p* = 0.004, lateral *p* = 0.009). On the medial side, the S group showed 9.30 MPa higher stress than the L group (95% CI: 0.55–18.06) and 8.87 MPa higher stress than the N group (95% CI: 2.59–15.15). On the lateral side, the S group had 5.81 MPa higher stress than the L group (95% CI: 0.64–10.98) and 5.56 MPa higher stress than the N group (95% CI: 1.18–9.93). No significant differences were found between the S and N groups (medial and lateral: *p* > 0.99) (Figure 9).

## 4. Discussion

Simulations were performed using the FEA cTHA model, showing that the average equivalent stress at the distal screw removal hole decreased as the stem length increased. The maximum equivalent stress at the distal screw removal hole showed significant differences between the S and L groups, as well as between the N and L groups, but no significant difference was found between the S and N groups. This is the first study to investigate in detail the impact of stem length on stress distribution around the distal screw removal hole in cTHA using FEA and statistical analysis.

Several studies have examined the relationship between cortical bone defects and bone strength using biomechanical techniques. In cadaver experiments, circular defects less than 20–30% of bone diameter had no significant effect on torsional strength [51,52]. Animal studies, such as those by Edgerton et al. on sheep femurs, showed no significant decrease in torsional strength with 10% defects, whereas 20% defects led to a 34% decrease, and a linear decrease in strength was observed with up to 60% defects [53]. Ho et al. found a 38% reduction in energy absorption with a 4-mm cortical defect in pig femurs [54]. Howieson et al. reported a 47% reduction in energy absorption with three 3.5-mm cortical defects in calves [55], and Brooks et al. showed a 55.2% reduction in energy absorption with 2.8-mm or 3.6-mm drill holes in dog femurs [56]. These studies suggest that following intramedullary nail removal, a 5-mm screw extraction hole in the femoral shaft may significantly reduce energy absorption, but its effect on torsional strength might not be as large.

The cortical bone defect should be bridged with a stem length at least twice the diameter of the femur (approximately 40 mm) [39,40,41]. Clinically, similar treatment strategies are often employed for stem-type fractures, such as Vancouver types B2 and B3, where the fracture site is bridged [40,41,57,58]. Several studies have biomechanically verified the bridging of cortical bone defects in femurs associated with cTHA, primarily reporting on the nail-plate fixation concept, where it was found that fracture did not occur even without a large stem bridging over screw extraction holes [33] and that a stem length of 1.5 × the femur diameter minimized stresses at the screw extraction site in cadaver experiments [59].

For continuous circumferential cortical defects such as fractures, sufficient strength, rigidity, and axial stability must be obtained via stem bridging. As previously mentioned, a bridging length that is at least twice the femoral diameter is recommended. However, for partial and smaller cortical bone defects such as those from implant removal holes, the impact on bone strength is limited, and the level of strength and stability required for fracture treatment is not necessary. The purpose of bridging in cTHA is to reinforce strength and rigidity against vulnerability from cortical bone defects and to reduce stress, and the required bridging length may be shorter than that for fractures or larger cortical bone defects.

Currently, a wide variety of stems and techniques are used in cTHA. However, no clinically significant method has been identified, and stem selection is largely at the surgeon’s discretion [7,11,34,35,37,38,60,61,62,63]. Furthermore, there are concerns regarding cement leakage and BCIS exist in cemented stems [61]. Several measures have been reported to prevent cement leakage, such as using gloves inflated with saline, bone wax, bone plugs made from the femoral head, and reinserting screws [33,64]. Generally, cement use is considered suitable for relatively older adult patients aged >70 years, especially in environments that are not conducive to osseointegration, such as poor bone quality or wide medullary cavities [8,34,63,65,66]. In contrast, many orthopedic surgeons prefer cementless stems, which provide more physiologically and biologically favorable fixation while avoiding cement leakage and BCIS [11,34,35,67]. Many surgeons use long stems to bridge screw extraction holes [11,34,35,37]. Good outcomes with cementless stems coated with hydroxyapatite over the entire circumference have also been reported in cTHA [57].

Structurally vulnerable areas such as screw extraction holes are prone to stress concentration, which increases the risk of fracture. Using a stem of insufficient length positioned close to the screw extraction site may lead to a hinge effect around this area, causing stress concentration. However, the insertion of a sufficiently long stem can distribute the load across a wide region of the femur. Because of its increased rigidity, this stem reduces the stress around the screw extraction hole by transferring the stress along the stem. The stem also potentially reinforces structural integrity around this weak point.

In this study, the distal screw length (femur diameter) averaged 25.7 ± 1.7 mm, with a screw diameter of 5 mm, equating to an approximately 20% bicortical bone defect. Based on biomechanical studies, this defect size may significantly reduce energy absorption, making it essential to bridge the distal screw extraction hole with a sufficiently long stem to reduce stress and prevent fractures.

In this study, the short stem (120 mm) overlapped the distal screw extraction hole, whereas the normal (130 mm) and long (160 mm) stems were bridged by approximately 10 mm and 40 mm, respectively. Bridging of 10 mm was insufficient to avoid stress concentration, whereas bridging of 40 mm (approximately 1.6 times the femoral cortex diameter) significantly reduced peak stress around the extraction hole. Therefore, the optimal stem length to reduce the maximum stress around the distal screw extraction hole is likely to be between 130 and 160 mm.

While long stems in cTHA have been associated with increased operative time, blood loss, and perioperative complications compared to standard stems [68], it is ideal to use a stem that meets the required bridging length. However, owing to the limited available stem options, revision of long stems may be necessary in practice. Special attention is required to prevent intraoperative fractures, and careful handling is required during surgery.

This study used FEA to simulate different stem lengths in cTHA models, providing a detailed analysis of the stress around the distal screw extraction hole, which has not been well understood previously. FEA allows stress measurement within the extraction hole, which is a challenge for traditional methods such as strain gauges or thermoelastic stress analysis. Additionally, using existing patient CT data, we reduced costs and ensured a sufficient sample size for statistical analysis. This enabled us to determine the stem length required to reduce the peak stress on the screw extraction hole.

Given that cortical bone defects may serve as points for stress accumulation, we believe that the observed significant reduction in maximum stress around screw holes with the use of a 160 mm long stem could have important clinical implications, thereby elevating the risk of fractures [8]. Specifically, it may contribute to mitigating the risk of screw hole-related fractures, which represent a notable concern in cTHA.

This study also has some limitations. First, this study was conducted under ideal conditions with bone and boundary conditions that differed from those in vivo. Specifically, the bone microstructure and dynamic load conditions were not fully represented in the model. We used MF software (version 13.0), which estimates bone density from CT values and allows FEA to incorporate the bone microstructure.

The static loading model in FEA has inherent limitations that restrict its ability to fully replicate real-world conditions. It fails to account for dynamic factors, such as time-dependent loading during activities like walking or impact, limiting its relevance for fatigue or vibration analysis. The simplification of boundary conditions often neglects the complex interactions between implants, bones, and surrounding tissues, reducing the model’s physiological accuracy. Nonlinear behaviors, including plastic deformation, friction, or material detachment, are frequently omitted, which can compromise the reliability of results in high-stress regions. Additionally, static models are constrained to single loading conditions, making them inadequate for evaluating cyclic loads or fatigue behavior over time.

Crucially, static models do not incorporate biological responses, such as bone remodeling or adaptation around implants, which are essential for long-term outcome predictions. While effective for assessing stress and deformation under specific conditions, static loading models require supplementary dynamic or fatigue analyses to capture the complexity of real-world biomechanical environments.

CT scanners play a critical role in determining the accuracy of FEA models. Higher-resolution scans not only capture precise geometric details and density distributions of bone, enhancing model fidelity but also increase data volume and computational cost. Lower resolutions, while more efficient, risk oversimplifying key structural features.

HU is vital for defining material properties, allowing the incorporation of bone density and regional variations. However, artifacts, particularly from metal implants, can introduce errors and require effective image processing for correction. Scan resolution also impacts mesh generation. Excessively fine meshes derived from high-resolution data can reduce computational efficiency, making it essential to balance detail and performance through appropriate simplifications tailored to the purpose of analysis. High-resolution scans are indispensable for analyzing intricate bone structures, while lower resolutions may suffice for broader stress distribution evaluations. Understanding and optimizing CT scanner settings based on analytical objectives is key to achieving accurate and efficient results.

Additionally, we modeled the highest load levels during daily activities based on previous studies, accounting for soft tissue dynamics to approximate real-life scenarios. Second, fracture lines, calluses formation, and bone sclerosis around the screw removal site were not included to simplify the model, which can lead to potentially underestimating the localized stress distribution and structural weakness. Building a model based on femur CT data after intramedullary nail insertion could represent fracture lines, callus formation, and sclerosis. However, metal artifacts complicate accurate CT value detection and stress analysis. Therefore, post-nail removal CT data are preferred, although cases involving only nail removal and subsequent CT scans are rare. Consequently, we reconstructed a nail extraction model by aligning 3D femur models with intramedullary nail STL data and substituting the nail’s physical properties with an “unused material” designation in MF. Third, we focused solely on stem length without considering width (canal fill ratio), shape, or fixation concepts. The selected #11 stem may not ideally match each patient’s medullary canal shape, although we controlled for the canal shape using the Dorr classification to select samples with similar canal morphology.

This study suggests that a sufficient bridging length for the screw extraction hole lies between 10 and 40 mm and recommends the use of long stems in cTHA following intramedullary nail surgeries. Future directions include further verification with 20 mm and 30 mm bridging lengths to determine the optimal bridging length. Additional studies using cemented stems for Dorr type B and C, as well as experimental and clinical trials, are needed to validate the FEA results with fracture strength testing.

## 5. Conclusions

In cTHA following intramedullary nailing for femoral trochanteric fractures, using a sufficiently long stem may reduce the stress concentration around the distal screw extraction holes, potentially lowering the risk of periprosthetic fractures. Selecting an appropriate stem length is crucial to minimize the risk of complications associated with cTHA.

## Figures and Tables

**Table 1:** Material parameters.

	Materials	Young’s Modulus [GPa]	Poisson’s Ratio
Femoral bone	Heterogeneous model	Keyak (1998) [46]	0.40
Stem	Titanium alloy (Ti-6Al-4V)	109	0.28

**Table 2:** Loading conditions.

Normal Walking					
Force	X (N)	Y (N)	Z (N)	Acting Point	%
Hip contact	Lt. 54.0/Rt. −54.0	32.8	−229.2	P0	238
ABD	Lt. 58.0/Rt. −58.0	4.3	86.5	P1	104
TFL-P	Lt. 7.2/Rt. −7.2	11.6	13.2	P1	19
TFL-D	Lt. −0.5/Rt. 0.5	−0.7	−19.0	P1	19
P1 total force	Lt. −64.7/Rt. 64.7	−15.2	80.7	P1	105
VL	Lt. −0.9/Rt. 0.9	−18.5	−92.9	P2	95
**Stair climbing**					
**Force**	**X (N)**	**Y (N)**	**Z (N)**	**Acting point**	**%**
Hip contact	Lt. 59.3/Rt. −59.3	60.6	−236.3	P0	251
ABD	Lt. 70.1/Rt. −70.1	28.8	84.9	P1	
ITT-P	Lt. 10.5/Rt. −10.5	3.0	12.8	P1	
ITT-D	Lt. −0.5/Rt. 0.5	−0.8	−16.8	P1	
TFL-P	Lt. 3.1/Rt. −3.1	4.9	2.9	P1	
TFL-D	Lt. −0.2/Rt. 0.2	−0.3	−6.5	P1	
P1 total force	Lt. −83.0/Rt. 83.0	−35.6	77.3	P1	119
VL	Lt. −2.2/Rt. 2.2	−22.4	−135.1	P2	137
VM	Lt. −8.8/Rt. 8.8	−39.6	−267.1	P3	270

ABD, Abductor; TFL, Tensor Fascia Latae; TFL-P, Tensor Fascia Latae-Proximal; TFL-D, Tensor Fascia Latae-Distal; VL, Vastus Lateralis; VM, Vastus Medialis.

**Table 3:** Patient characteristics and 3D-cTHA model.

Characteristic	Value
Age (mean ± SD) [years]	66.5 ± 8.7
Hight (mean ± SD) [m]	1.53 ± 0.06
Body weight (mean ± SD) [kg]	53.5 ± 9.0
Body mass index (mean ± SD) [kg/m^2^]	22.7 ± 3.2
Side [limb]	Left 16; Right 14
Bone mineral density of the femoral neck (mean ± SD) [g/cm^2^]	0.61 ± 0.13
Canal flare index (mean ± SD)	4.17 ± 0.42
Length of distal screw (mean ± SD) [mm]	25.7 ± 1.7
Femoral anteversion (mean ± SD) [degrees]	21.69 ± 10.69

## Data Availability

The data are available from the corresponding author if required.
